# Association Between Copper and Global Cognition and the Moderating Effect of Iron

**DOI:** 10.3389/fnagi.2022.811117

**Published:** 2022-03-29

**Authors:** Young Min Choe, Guk-Hee Suh, Boung Chul Lee, Ihn-Geun Choi, Jun Ho Lee, Hyun Soo Kim, Jee Wook Kim

**Affiliations:** ^1^Department of Neuropsychiatry, Hallym University Dongtan Sacred Heart Hospital, Hwaseong, South Korea; ^2^Department of Psychiatry, Hallym University College of Medicine, Chuncheon, South Korea; ^3^Department of Neuropsychiatry, Hallym University Hangang Sacred Heart Hospital, Seoul, South Korea; ^4^Department of Psychiatry, Seoul W Psychiatric Office, Seoul, South Korea; ^5^Department of Neuropsychiatry, Seoul National University Hospital, Seoul, South Korea; ^6^Department of Laboratory Medicine, Hallym University Dongtan Sacred Heart Hospital, Hwaseong, South Korea

**Keywords:** copper, iron, MMSE, global cognition, Alzheimer’s disease

## Abstract

**Background:**

Despite the known association between abnormal serum copper levels and Alzheimer’s disease (AD) or cognitive decline, the association between copper, iron, and cognition remains poorly investigated. We examined the association between serum copper levels and global cognition measured using the Mini-Mental State Examination (MMSE) in older adults with normal copper levels. We also explored the moderating effect of iron on this association.

**Methods:**

The study enrolled 99 non-demented adults between 65 and 90 years of age. All the participants underwent comprehensive clinical assessments and serum copper measurements. Global cognitive performance was measured by the MMSE. All copper levels were within the normal range and were stratified into three categories: < 87 (low), 87–98 (medium), and > 98 (high: used as a reference category) μg/dL.

**Results:**

Serum copper level (as a continuous variable) was significantly associated with MMSE score (*B* = 0.065, 95% confidence interval = 0.023–0.108, *p* = 0.003). Low serum copper group showed significantly decreased MMSE score compared to high copper one (*B* = −2.643, 95% confidence interval = −4.169 to -1.117, *p* < 0.001), while middle copper category had no difference (*B* = −1.211, 95% confidence interval = −2.689 to 0.268, *p* = 0.107). There was a significant low serum copper ×iron interaction effect on the MMSE score (*B* = 0.065, 95% confidence interval = 0.016–0.114, *p* = 0.010). Subgroup analyses showed that low serum copper was significantly associated with a low MMSE score in the low-iron (*B* = −4.174, 95% confidence interval = −6.607 to −1.741, *p* = 0.001) but not high-iron subgroup (*B* = −0.721, 95% confidence interval = −2.852 to 1.409, *p* = 0.495).

**Conclusion:**

Our findings from non-demented older adults suggest that a low serum copper level within the normal range was associated with AD or cognitive decline and this is moderated by iron. To prevent AD or cognitive decline, clinicians need to pay attention to avoiding low serum copper and iron levels, even within the clinical normal range.

## Introduction

Plenty of evidence has indicated a relationship between food or nutritional intake, one of the most modifiable lifestyle factors, and the risk of Alzheimer’s disease (AD) and cognitive decline (Solfrizzi et al., 2003; Morris and Tangney, 2010; Jeon et al., 2019). While some evidence has focused on macronutrients components as AD and cognitive decline risk factors, (Solfrizzi et al., 2003; Gu et al., 2010; Berti et al., 2018) other evidence suggested an association between micronutrients including minerals and AD or related cognitive decline (Bush, 2003; Solfrizzi et al., 2003; Bush and Strozyk, 2004; Vaz et al., 2018; Squitti et al., 2019; Kim et al., 2021).

Among the micronutrients, the relationship between copper and AD has been hotly debated topic in the field of neurology as researchers attempt to find a cure for the AD condition that affect about 24 million people worldwide (Mayeux and Stern, 2012). Copper is the third most abundant trace metal element in the bran and is essential to human life (Scheiber et al., 2014). However, it is very likely to be involved in AD and cognitive decline (Squitti et al., 2006) and its role in AD and related cognitive dysfunction is complex (Li et al., 2017; Squitti et al., 2021). Preclinical studies have indicated that excessive copper levels are associated with beta-amyloid protein (Aβ) in AD with metal imbalance, (White et al., 1999; Opazo et al., 2002) and human studies found similar results in poor cognitive performance (Oder et al., 1993; Smorgon et al., 2004; Li et al., 2017). In contrast, a couple of studies indicated the positive association between copper level and cognitive performance under conditions below the inflection point of dietary copper intake (Wang et al., 2021) or excluding the highest abnormal blood copper level (Pajonk et al., 2005). Taken together, it is important to investigate the effects of copper on cognitive performance without abnormal copper level in order to understand the precise effects of copper on cognitive function in older adults with normal copper level. In the normal state, a more pronounced association between copper levels and cognitive performance could be observed in older adults apart from the copper toxicity in AD and related cognitive decline with abnormal copper levels (Oder et al., 1993; White et al., 1999; Opazo et al., 2002; Smorgon et al., 2004). In addition, brain iron as well as copper homeostasis is important because copper and iron lead to neuropathology on AD (Madsen and Gitlin, 2007; Collins et al., 2010; Ayton et al., 2021). Therefore, proper regulation of both copper and iron via interactions between two is critical to avoid AD and related cognitive decline with metal imbalance (Madsen and Gitlin, 2007; Collins et al., 2010; Ayton et al., 2021).

Therefore, we examined that association between serum copper levels and global cognition using the mini-mental state examination (MMSE) (Nakata et al., 2009) in older adults with normal copper levels. We also explored the moderating effect of iron on the association between serum copper levels and global cognition.

## Materials and Methods

### Participants

This study is part of the General Lifestyle and AD (GLAD) study, which is an ongoing prospective cohort study that began in 2020. As of August 2021, in total, 112 individuals had volunteered for the assessment of eligibility for the GLAD study (Supplementary Figure 1). Among them, 99 non-demented adults between 65 and 90 years of age: 42 cognitively normal (CN) adults and 57 adults with mild cognitive impairment (MCI) were enrolled in the GLAD study and 13 individuals were excluded due to withdrawal of consent (*n* = 12) or loss of content (*n* = 1). Participants were recruited from individuals who participated in a dementia screening program at the memory clinic of Hallym University Dongtan Sacred Heart Hospital, Hwaseong, South Korea. Those who volunteered were invited for an eligibility assessment. In addition, volunteers from the community were recruited through recommendations from other participants, family members, friends, or acquaintances. The CN group consisted of participants with a Clinical Dementia Rating (Morris, 1993) score of 0 and no diagnosis of MCI or dementia. All participants with MCI met the current consensus criteria for amnestic MCI, including: memory complaints confirmed by an informant; objective memory impairment; preservation of global cognitive function; independence in functional activities; and absence of dementia. Regarding objective memory impairment, the age-, education-, and sex-adjusted z-score was < −1.0 for at least one of four episodic memory tests included in the Korean version of the Consortium to Establish a Registry for Alzheimer’s Disease (CERAD-K) neuropsychological battery: word list memory, word list recall, word list recognition, and constructional recall tests (Morris et al., 1989; Lee et al., 2002, 2004). All MCI individuals had a Clinical Dementia Rating score of 0.5. The exclusion criteria were the presence of a major psychiatric illness or a significant neurological or medical condition or comorbidity that could affect mental functioning; illiteracy; the presence of visual/hearing difficulties and/or severe communication or behavioral problems that would make clinical examinations difficult; and use of an investigational drug. This study protocol was approved by the institutional review board of the Hallym University Dongtan Sacred Heart Hospital and was conducted it in accordance with the recommendations of the current version of the Declaration of Helsinki. The subjects or their legal representatives gave informed consent.

### Clinical Assessments

All participants underwent standardized clinical assessments by trained psychiatrists based on the GLAD study clinical assessment protocol, which incorporated the Korean version of the CERAD-K (Morris et al., 1989; Lee et al., 2002). Trained neuropsychologists also administered the GLAD neuropsychological assessment protocol incorporating the CERAD-K neuropsychological battery (Lee et al., 2004) to all participants. The MMSE (Nakata et al., 2009) was used to measure global cognitive function. Vascular risks (e.g., hypertension, diabetes mellitus, dyslipidemia, coronary heart disease, transient ischemic attack, and stroke) were assessed based on data collected by trained researchers during systematic interviews of participants and their family members. A vascular risk score (VRS) was calculated based on the number of vascular risk factors present and reported as a percentage (DeCarli et al., 2004). The Geriatric Depression Scale (GDS) was used to measure the severity of depressive symptoms (Yesavage et al., 1982; Kim et al., 2008). Annual income was categorized into below the minimum cost of living (MCL), more than the MCL but below twice the MCL, and twice the MCL or more.^1^ The MCL was determined from data published by the Ministry of Health and Welfare, Republic of Korea in November 2012 and was 572,168 Korean Won (KRW) [equivalent to 507.9 US dollars (USD)] per month for single-person households with an additional 286,840 KRW (equivalent to 254.6 USD) per month for each additional housemate. To ensure that the information was accurate, reliable informants were also interviewed. Lifetime alcohol intake status (never/former/drinker) and smoking status (never/ex-smoker/smoker) were also evaluated through trained researcher interviews and a medical record review. Information about nutritional state, including the change in food intake over the past 3 months due to loss of appetite, digestive problems, and chewing or swallowing difficulties was obtained using the mini nutritional assessment (MNA) tool (Vellas et al., 1999). To acquire accurate information, reliable informants were interviewed.

### Measuring Serum Copper Levels and Other Blood Biomarkers

After an overnight fast, blood samples were obtained by venipuncture in the morning (8–9 a.m.). The serum copper level was measured using an ELAN DRC-e inductively coupled plasma-mass spectrometer (Perkin Elmer, United States). The normal range for the serum copper level is 75–145 μg/dL according to the Mayo Clinic Laboratories.^2^ Based on tertiles of the serum copper level, participants were categorized in low (< 87 μg/dL), medium (87–98 μg/dL), and high (> 98 μg/dL) (used as the reference category) level groups. Albumin, glucose, high-density lipoprotein (HDL)-cholesterol, low-density lipoprotein (LDL)-cholesterol, and iron were measured using a COBAS c702 analyzer and dedicated reagents (Roche Diagnostics, Germany). Based on the median quartile of iron levels, participants were also categorized into two groups (high iron group *vs.* low iron group). The hemoglobin level was determined using an XN-3000 automated hematology analyzer and dedicated reagents (Sysmex, Japan). Apolipoprotein E (apoE) was genotyped using the Seeplex ApoE ACE genotyping kit (Seegene, South Korea). ApoE ε4 allele (APOE4)-positivity was defined as the presence of at least one ε4 allele.

### Statistical Analysis

To examine the relationships between the serum copper and global cognition, multiple linear regression analyses with copper (continuous variables or categorical variables, i.e., low copper, medium copper, and high copper) as the independent variable and global cognition as the dependent variable were performed. The association between serum copper and global cognition may be influenced by various factors. Therefore, we systematically evaluated all participants to identify potential confounders, such as vascular risks, depression, annual income, alcohol intake, smoking, blood markers including albumin, hemoglobin, glucose, HDL- and LDL-cholesterol, and iron. We tested three models, controlling for the covariates in a stepwise manner. The first model included age, sex, APOE4, and education as covariates; the second model included those covariates plus clinical diagnosis, VRS, GDS, annual income status, alcohol intake, and smoking; and, the third model included the covariates in the second model plus albumin, hemoglobin, glucose, HDL- and LDL-cholesterol, and iron. As sensitivity analyses, the same analyses were performed for the subjects with cognitive impairment because MMSE may be less discriminatory in the CN individuals. The same analyses were also performed for the subjects with no decrease in food intake over the past 3 months for any reason to eliminate any influence of physical or mental condition, which can potentially relate to both serum copper levels and cognition or brain status. To explore the moderating effects of age, sex, APOE4-positivity, VRS, and iron levels on the associations between serum copper levels and global cognition that were significant in the analyses described above, the regression analyses were repeated including two-way interaction terms between serum copper levels and the biomarkers as additional independent variables. All statistical analyses were performed using SPSS Statistics software ver. 27 (IBM, Armonk, NY, United States).

## Results

### Participants

Table 1 summarized the demographic and clinical characteristics of the overall participants. All participants had serum copper levels within normal range. Of the 99 participants, 32, 35, and 32 had low, medium, and high copper levels, respectively. Supplementary Tables 1, 2 summarized the demographic and clinical characteristics of CN and MCI, respectively.

**TABLE 1:** Demographic and clinical characteristics of non-demented participants by the categories of serum copper.

Characteristic	Overall	Categorized copper level			*P*
		Low	Medium	High	
*n*	99	32	35	32	
Age, y	72.25 (5.35)	72.44 (4.41)	72.54 (6.20)	71.75 (5.33)	0.812*^a^*
Female, *n* (%)	76 (76.77)	25 (78.13)	27 (77.14)	24 (75.00)	0.955*^b^*
Education, y	8.76 (4.62)	8.50 (4.13)	9.31 (4.76)	8.406 (5.01)	0.677*^a^*
APOE4-positivity, *n* (%)	19 (19.19)	9 (28.13)	5 (14.29)	5 (15.63)	0.293*^b^*
MCI, *n* (%)	57 (57.58)	21 (65.63)	16 (45.71)	20 (62.50)	0.204*^b^*
VRS, %	16.67 (17.50)	18.75 (18.33)	15.71 (17.59)	15.62 (16.90)	0.719*^a^*
GDS score	11.00 (7.22)	9.50 (6.17)	10.77 (6.98)	12.75 (8.22)	0.193*^a^*
**Annual income status**					0.172*^b^*
< MCL, *n* (%)	18 (18.18)	8 (25.00)	2 (5.71)	8 (25.00)	
≥MCL, < 2 ×MCL, *n* (%)	30 (30.30)	11 (34.38)	11 (31.43)	8 (25.00)	
≥2 ×MCL, *n* (%)	50 (50.50)	13 (40.63)	21 (60.00)	16 (50.00)	
**Alcohol drink status, *n* (%)**					0.940*^b^*
Never	53 (53.53)	18 (56.25)	19 (54.29)	16 (50.00)	
Former	20 (20.20)	6 (18.75)	8 (22.86)	6 (18.75)	
Drinker	26 (26.26)	8 (25.00)	8 (22.86)	10 (31.25)	
**Smoking status, *n* (%)**					0.553*^c^*
Never	78 (78.78)	25 (78.13)	27 (77.14)	26 (81.25)	
Former	18 (18.18)	6 (18.75)	8 (22.86)	4 (0.125)	
Smoker	3 (3.03)	1 (3.13)	0 (0.00)	2 (6.25)	
Copper, μg/dL	95.39 (14.79)	80.78 (3.48)	90.86 (3.57)	112.78 (11.22)	<0.001*^a^*
Hemoglobin, g/dL	13.31 (1.63)	13.03 (1.71)	13.33 (1.83)	13.58 (1.27)	0.412*^a^*
Albumin, g/dL	4.59 (0.25)	4.62 (0.23)	4.62 (0.27)	4.53 (0.26)	0.247*^a^*
Glucose, fasting, mg/dL	112.96 (24.00)	110.75 (17.64)	112.51 (25.82)	115.66 (27.68)	0.713*^a^*
HDL-cholesterol, mg/dL	55.07 (12.88)	53.97 (12.71)	57.43 (14.31)	53.59 (11.33)	0.405*^a^*
LDL-cholesterol, mg/dL	97.58 (33.45)	89.16 (25.82)	100.91 (31.88)	102.34 (40.59)	0.222*^a^*
Iron, μg/dL	100.86 (33.07)	98.16 (26.02)	103.51 (33.82)	100.66 (38.86)	0.806*^a^*
Iron					0.830*^b^*
High (≥100 μg/dL), *n* (%)	49 (49.49)	17 (53.13)	16 (45.71)	16 (50.00)	
Low (<100 μg/dL), *n* (%)	50 (50.50)	15 (46.88)	19 (54.29)	16 (50.00)	
Decrease in food intake over the past 3 months					0.820*^c^*
no, *n* (%)	14 (14.14)	4 (12.50)	6 (17.14)	4 (12.50)	
yes, *n* (%)	85 (85.85)	28 (87.5)	29 (82.86)	28 (87.50)	
Global cognitive performance					
MMSE raw score	25.35 (3.78)	23.63 (4.25)	26.00 (3.01)	26.38 (3.55)	0.006*^a^*
MMSE z- score	0.04 (1.15)	−0.54(1.31)	0.18 (0.99)	0.46 (0.90)	0.001*^a^*

*APOE4, apolipoprotein E ε4 allele; MCI, mild cognitive impairment; VRS vascular risk score; GDS, geriatric depression scale; MCL minimum cost of living; MMSE, mini-mental state examination.*

*Data are expressed as mean (standard deviation), unless otherwise indicated.*

*^a^By one-way analysis of variance.*

*^b^By chi-square test.*

*^c^By fisher exact test.*

### Association of the Serum Copper Level With Global Cognition

**FIGURE 1:**
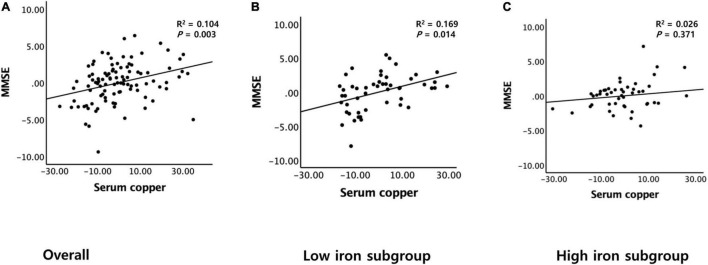
Partial regression plots of the serum copper and MMSE score. **(A)** Overall and **(B,C)** by subgroup (**B**, low iron and **C**, high iron). Multiple linear regression analyses were performed after adjusting for all confounders.

**FIGURE 2:**
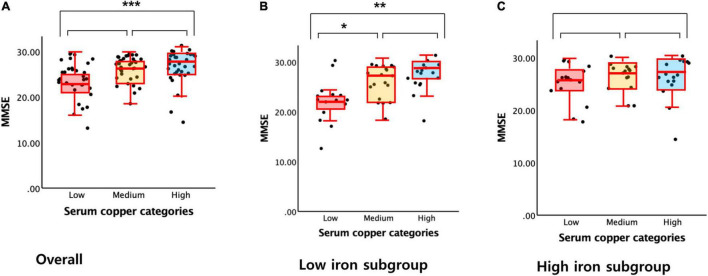
Box plots of the serum copper categories and MMSE score. **(A)** Overall and **(B,C)** by subgroup (**B**, low iron and **C**, high iron). Multiple linear regression analyses were performed after adjusting for all confounders. *P < 0.05; **P < 0.01; ***P < 0.001.

A serum copper level (as a continuous variable) was significantly associated with MMSE score (*B* = 0.065, 95% confidence interval = 0.023–0.108, *p* = 0.003) (Table 2 and Figure 1A). A low serum copper category was also significantly associated with decreased MMSE score compared to a high serum copper (*B* = −2.643, 95% confidence interval = −4.169 to -1.117, *p* < 0.001), while middle copper category had no difference (*B* = −1.211, 95% confidence interval = −2.689 to 0.268, *p* = 0.107) (Table 3 and Figure 2A). Sensitivity analysis of the participants with MCI and those with no decrease in food intake over the past 3 months gave similar results for MMSE (Tables 4–10).

**TABLE 2:** The results of multiple linear regression analyses assessing the associations between the serum copper level and MMSE in non-demented older adults.

	B (SE)	CI	*P*	Adjusted *R*^2^
Model 1	0.056 (0.022)	0.012–0.101	0.014	0.282
Model 2	0.061 (0.021)	0.019–0.103	0.005	0.449
Model 3	0.065 (0.021)	0.023–0.108	0.003	0.447

*MMSE, mini-mental state examination; APOE4 apolipoprotein E ε4 allele; VRS vascular risk score; GDS, geriatric depression scale.*

*Model 1 included age, sex, APOE4-positivity, and education; model 2 included age, sex, APOE4-positivity, education, clinical diagnosis, VRS, GDS score, annual income status, alcohol, and smoking; and model 3 included age, sex, APOE4-positivity, education, clinical diagnosis, VRS, GDS score, annual income status, alcohol, smoking, albumin, hemoglobin, glucose, HDL-cholesterol, LDL-cholesterol, and iron.*

**TABLE 3:** The results of multiple linear regression analyses assessing the associations between the serum copper strata and MMSE in non-demented older adults.

	Low			Medium			High	Adjusted *R*^2^
	B (SE)	CI	*P*	B (SE)	CI	*P*		
Model 1	−2.804 (0.789)	−4.371 to −1.238	<0.001	−0.688 (0.773)	−2.224 to 0.847	0.375	Reference	0.326
Model 2	−2.444 (0.755)	−3.945 to −0.943	0.002	−1.064 (0.718)	−2.492 to 0.365	0.142	Reference	0.456
Model 3	−2.643 (0.766)	−4.169 to −1.117	<0.001	−1.211 (0.766)	−2.689 to 0.268	0.107	Reference	0.457

*MMSE, mini-mental state examination; APOE4 apolipoprotein E ε4 allele; VRS vascular risk score; GDS, geriatric depression scale.*

*Model 1 included age, sex, APOE4-positivity, and education; model 2 included age, sex, APOE4-positivity, education, clinical diagnosis, VRS, GDS score, annual income status, alcohol, and smoking; and model 3 included age, sex, APOE4-positivity, education, clinical diagnosis, VRS, GDS score, annual income status, alcohol, smoking, albumin, hemoglobin, glucose, HDL-cholesterol, LDL-cholesterol, and iron.*

**TABLE 4:** The results of multiple linear regression analyses assessing the associations between the serum copper level and MMSE in MCI older adults.

	B (SE)	CI	*P*	Adjusted *R*^2^
Model 1	0.074 (0.026)	0.020–0.127	0.008	0.403
Model 2	0.081 (0.031)	0.020–0.143	0.011	0.397
Model 3	0.087 (0.032)	0.022–0.151)	0.010	0.433

*MMSE, mini-mental state examination; APOE4 apolipoprotein E ε4 allele; VRS vascular risk score; GDS, geriatric depression scale.*

*Model 1 included age, sex, APOE4-positivity, and education; model 2 included age, sex, APOE4-positivity, education, VRS, GDS score, annual income status, alcohol, and smoking; and model 3 included age, sex, APOE4-positivity, education, clinical diagnosis, VRS, GDS score, annual income status, alcohol, smoking, albumin, hemoglobin, glucose, HDL-cholesterol, LDL-cholesterol, and iron.*

**TABLE 5:** The results of multiple linear regression analyses assessing the associations between the serum copper strata and MMSE in MCI older adults.

	Low			Medium			High	Adjusted *R*^2^
	B (SE)	CI	*P*	B (SE)	CI	*P*		
Model 1	−3.209 (1.039)	−5.295 to −1.123	0.003	−1.418 (1.101)	−3.630 to 0.794	0.204	Reference	0.412
Model 2	−3.461 (1.180)	−5.837 to −1.085	0.005	−1.716 (1.206)	−4.145 to 0.712	0.161	Reference	0.402
Model 3	−3.915 (1.313)	−6.571 to −1.258	0.005	−2.785 (1.418)	−5.652 to 0.002	0.057	Reference	0.440

*MMSE, mini-mental state examination; APOE4 apolipoprotein E ε4 allele; VRS vascular risk score; GDS, geriatric depression scale.*

*Model 1 included age, sex, APOE4-positivity, and education; model 2 included age, sex, APOE4-positivity, education, VRS, GDS score, annual income status, alcohol, and smoking; and model 3 included age, sex, APOE4-positivity, education, clinical diagnosis, VRS, GDS score, annual income status, alcohol, smoking, albumin, hemoglobin, glucose, HDL-cholesterol, LDL-cholesterol, and iron.*

**TABLE 6:** The results of multiple linear regression analyses assessing the associations between the serum copper level and MMSE in non-demented older adults with no decrease in food intake over the past 3 months (*n* = 85).

	B (SE)	CI	*P*	Adjusted *R*^2^
Model 1	0.060 (0.024)	0.012–0.107	0.014	0.323
Model 2	0.073 (0.023)	0.028–0.118	0.002	0.472
Model 3	0.076 (0.023)	0.029–0.122	0.002	0.459

*MMSE, mini-mental state examination; APOE4 apolipoprotein E ε4 allele; VRS vascular risk score; GDS, geriatric depression scale.*

*Model 1 included age, sex, APOE4-positivity, and education; model 2 included age, sex, APOE4-positivity, education, clinical diagnosis, VRS, GDS score, annual income status, alcohol, and smoking; and model 3 included age, sex, APOE4-positivity, education, clinical diagnosis, VRS, GDS score, annual income status, alcohol, smoking, albumin, hemoglobin, glucose, HDL-cholesterol, LDL-cholesterol, and iron.*

**TABLE 7:** The results of multiple linear regression analyses assessing the associations between the serum copper strata and MMSE in non-demented older adults with no decrease in food intake over the past 3 months (*n* = 85).

	Low			Medium			High	Adjusted *R*^2^
	B (SE)	CI	*P*	B (SE)	CI	*P*		
Model 1	−2.775 (0.828)	−4.424 to −1.126	0.001	−0.893 (0.818)	−2.522 to 0.736	0.278	Reference	0.357
Model 2	−2.764 (0.799)	−4.356 to −1.171	<0.001	−1.394 (0.784)	−2.957 to 0.168	0.079	Reference	0.477
Model 3	−3.055 (0.825)	−4.703 to −1.407	<0.001	−1.515 (0.802)	−3.118 to 0.087	0.063	Reference	0.473

*MMSE, mini-mental state examination; APOE4 apolipoprotein E ε4 allele; VRS vascular risk score; GDS, geriatric depression scale.*

*Model 1 included age, sex, APOE4-positivity, and education; model 2 included age, sex, APOE4-positivity, education, clinical diagnosis, VRS, GDS score, annual income status, alcohol, and smoking; and model 3 included age, sex, APOE4-positivity, education, clinical diagnosis, VRS, GDS score, annual income status, alcohol, smoking, albumin, hemoglobin, glucose, HDL-cholesterol, LDL-cholesterol, and iron.*

**TABLE 8:** The results of multiple linear regression analyses including interaction terms between the serum copper strata and age (or sex, or APOE4-positivity, or VRS, or iron) in terms of predicting MMSE.

	B (SE)	CI	*P*
Low copper	−18.138 (11.187)	−40.413 to 4.137	0.109
Medium copper	−16.714 (9.420)	−35.471 to 2.043	0.080
Age	−0.159 (0.102)	−0.362 to 0.043	0.122
Low copper ×Age	0.216 (0.155)	−0.093 to 0.524	0.168
Medium copper ×Age	0.215 (0.130)	−0.044 to 0.473	0.102
Low copper	−3.121 (0.905)	−4.923 to −1.320	< 0.001
Medium copper	−1.344 (0.893)	−3.121 to 0.433	0.136
Sex	−2.086 (1.715)	−5.500 to 1.329	0.228
Low copper × Sex	2.015 (1.891)	−1.750 to 5.780	0.290
Medium copper × Sex	0.674 (1.919)	−3.148 to 4.495	0.727
Low copper	−2.876 (0.899)	−4.665 to −1.086	0.002
Medium copper	−1.254 (0.854)	−2.954 to 0.446	0.146
APOE4 positivity	−0.045 (1.680)	−3.391 to 3.301	0.979
Low copper ×APOE4-positivity	0.994 (2.029)	−3.046 to 5.033	0.626
Medium copper ×APOE4-positivity	0.149 (2.298)	−4.427 to 4.724	0.949
Low copper	−2.592 (1.510)	−5.599 to 0.415	0.090
Medium copper	−1.721 (1.248)	−4.206 to 0.765	0.172
VRS	−0.031 (0.037)	−0.105 to 0.043	0.402
Low copper ×VRS	0.002 (0.051)	−0.100 to 0.103	0.969
Medium copper × VRS	0.022 (0.045)	−0.068 to 0.112	0.631
Low copper	−9.075 (2.562)	−14.176 to −3.974	< 0.001
Medium copper	−3.382 (2.148)	−7.659 to 0.895	0.119
Iron	−0.019 (0.014)	−0.047 to 0.009	0.175
Low copper×Iron	0.065 (0.025)	0.016 to 0.114	0.010
Medium copper ×Iron	0.023 (0.020)	0.018 to 0.063	0.268

*MMSE, mini-mental state examination; APOE4 apolipoprotein ε4; VRS vascular risk score; APOE4 apolipoprotein E ε4 allele.*

*Multiple linear regression model included copper, age (or sex, or APOE4-positivity, or VRS, or iron) and the interaction between copper and age (or sex, or APOE4-positivity, or VRS, or iron) treated as the independent variables; for all potential confound factors were treated as covariates; and MMSE treated as the dependent variable.*

**TABLE 9:** The results of multiple linear regression analyses assessing the associations between the serum copper level and MMSE by iron subgroup.

	B (SE)	CI	*P*	Adjusted *R*^2^
**High iron** (*n* = 49)				
Model 1	0.015 (0.029)	−0.043 to 0.073	0.604	0.360
Model 2	0.029 (0.030)	−0.032 to 0.090	0.344	0.434
Model 3	0.028 (0.031)	−0.035 to 0.092	0.371	0.440
**Low iron** (*n* = 50)				
Model 1	0.092 (0.035)	0.021 to 0.163	0.012	0.233
Model 2	0.082 (0.031)	0.019 to 0.145	0.012	0.488
Model 3	0.087 (0.033)	0.019 to 0.155	0.014	0.479

*MMSE, mini-mental state examination; APOE4 apolipoprotein E ε4 allele; VRS vascular risk score; GDS, geriatric depression scale.*

*Model 1 included age, sex, APOE4-positivity, and education; model 2 included age, sex, APOE4-positivity, education, clinical diagnosis, VRS, GDS score, annual income status, alcohol, and smoking; and model 3 included age, sex, APOE4-positivity, education, clinical diagnosis, VRS, GDS score, annual income status, alcohol, smoking, albumin, hemoglobin, glucose, HDL-cholesterol, and LDL-cholesterol.*

**TABLE 10:** The results of multiple linear regression analyses assessing the associations between the serum copper strata and MMSE by iron subgroup.

	Low			Medium			High	Adjusted R^2^
	B (SE)	CI	*P*	B (SE)	CI	*P*		
**High iron (*n* = 49)**								
Model 1	−0.740 (1.013)	−2.786 to 1.306	0.469	−0.104 (1.017)	−2.158 to 1.950	0.919	Reference	0.350
Model 2	−0.948 (1.002)	−2.983 to 1.087	0.351	−0.443 (1.091)	−2.657 to 1.772	0.687	Reference	0.418
Model 3	−0.721 (1.043)	−2.852 to 1.409	0.495	−0.934 (1.136)	−3.254 to 1.386	0.418	Reference	0.421
**Low iron (*n* = 50)**								
Model 1	−5.034 (1.210)	−7.474 to −2.595	< 0.001	−1.173 (1.112)	−3.417 to 1.070	0.297	Reference	0.368
Model 2	−3.977 (1.139)	−6.285 to −1.669	0.001	−1.442 (0.974)	−3.416 to 0.531	0.147	Reference	0.532
Model 3	−4.174 (1.194)	−6.607 to −1.741	0.001	−1.236 (1.062)	−3.398 to 0.927	0.253	Reference	0.540

*MMSE, mini-mental state examination; APOE4 apolipoprotein E ε4 allele; VRS vascular risk score; GDS, geriatric depression scale.*

*Model 1 included age, sex, APOE4-positivity, and education; model 2 included age, sex, APOE4-positivity, education, clinical diagnosis, VRS, GDS score, annual income status, alcohol, and smoking; and model 3 included age, sex, APOE4-positivity, education, clinical diagnosis, VRS, GDS score, annual income status, alcohol, smoking, albumin, hemoglobin, glucose, HDL-cholesterol, and LDL-cholesterol.*

### Moderation of the Association Between the Serum Copper and Global Cognition

The serum copper × iron interaction was significant in terms of the MMSE score, indicating that iron moderates the association between the serum copper and global cognition (*B* = 0.065, 95% confidence interval = 0.016–0.114, *p* = 0.010) (Table 8). Further subgroup analyses showed that the association between a low serum copper level and decreased MMSE score was present only in older adults with low iron group (*B* = −4.174, 95% confidence interval = −6.607 to -1.741, *p* = 0.001), and not in those with high iron group (*B* = −0.721, 95% confidence interval = −2.852 to 1.409, *p* = 0.495) (Table 8 and Figures 1B,C, 2B,C). The interactions between the serum copper and age, sex, APOE-4 positivity, and VRS were not significant (Table 8).

## Discussion

This study of non-demented adults with normal copper levels showed that low serum copper was associated with a worse global score. The present findings consistent with results of previous human studies regarding the association between serum copper level and cognitive decline or AD dementia. The National Health and Nutrition Examination Surveys indicated the nonlinear association between copper intake and cognitive performance. They revealed that higher copper intake was associated with better cognitive performance when copper intake was below the inflection point, but not in above inflection point (Wang et al., 2021). Furthermore, a study of AD subjects supported our findings in that plasma copper was positively associated with cognitive performance when excluding those with the highest tertile of copper (i.e., 133–165 μg/dL) (Pajonk et al., 2005). These are similar to those obtained after excluding abnormal copper levels above 145 μg/dL in our study. This suggests that high copper level within normal ranges did not show copper toxicity and protects cognitive decline. Furthermore, low copper level affects cognitive decline even in the normal range of copper. As their study is limited to AD dementia subjects, (Pajonk et al., 2005) the association between copper and cognitive performance may not be clear in the preclinical stage of AD dementia. In this point, our study has a novelty finding in that the copper-global cognition association may appear from the preclinical stage of AD dementia different from their findings from AD dementia.

We also found that iron status moderated the association between the serum copper level and MMSE score. There was a significant association between low copper level and worse MMSE performance in participants with low iron levels, but not in those with high iron levels. This may reflect associative interactions between copper, iron, and cognitive decline. Brain copper and iron homeostasis is carefully regulated by a series of influx and efflux transporters because, while essential, excess copper and iron lead to neuropathology (Madsen and Gitlin, 2007; Collins et al., 2010). The regulation of both copper and iron is critical to avoid neurological disturbances associated with metal imbalance in AD (Madsen and Gitlin, 2007; Collins et al., 2010). The interaction between copper and iron may be important for regulating copper–iron homeostasis, in line with our findings.

Regarding the mechanism underlying the relationship between lower serum copper and AD and related cognitive decline, an imbalance of metal-ion homeostasis in the brain is thought to play an important role in the pathogenesis of AD. Copper was bound to Aβ plaques and remained high in senile plaques in an AD mouse model (James et al., 2017) and such plaques were markedly enriched with copper in the human brain (Lovell et al., 1998; Cherny et al., 1999). Lower serum copper may reflect sequestration of copper in the brain due to its binding to Aβ and depletion in other body compartment such as blood. Although the consequence of copper sequestration by Aβ peptides and its impact on AD pathogenesis is not clearly understood yet, recent evidence suggest that decreased copper levels in the synaptic cleft can alter glutamatergic excitotoxic neurotransmission and promote synaptic failure and neuronal death (Opazo et al., 2014; Sensi et al., 2018). Alternatively, lower serum copper level caused by dietary copper deficiency might result in AD or worse cognition, as has been demonstrated in an animal model study (Bayer et al., 2003). However, this possibility seems not so high given that the sensitivity analysis for individuals with no nutritional deficiency revealed similar results.

## Strengths and Limitations

To the best of our knowledge, this study is the first to show a positive association between the serum copper level and global cognition in non-demented old adults with normal copper levels (adjusted *R*^2^ = 0.45, *B* = 0.065, 95% confidence interval = 0.023–0.108, *p* = 0.003), as well as the moderating effect of low iron on the association (adjusted *R*^2^ = 0.48, *B* = 0.087, 95% confidence interval = 0.019–0.155, *p* = 0.014). In addition, the findings are maintained even after adjusting for various potential confounders, for example, biological, environmental, and hematological biomarkers. The results were also confirmed by sensitivity analysis performed after excluding participants who were CN or those with decreased food intake. Our study had a couple of limitations. First, as it was a cross-sectional study, we could not confirm a causal relationship between chronic copper status in blood and cognitive decline. As severe medical condition including dementia and poor nutrition may affect copper and cognition, we tried to minimize such reverse causality. We excluded individuals with severe medical conditions that could affect mental function. Demented patients commonly had a poor nutrition which may reduce serum copper level. This may explain the associations between low serum copper and AD dementia and related cognitive decline reported previously (Pajonk et al., 2005). As all of our participants were non-demented, however, reverse causality could not explain our observation of the relationship between low copper and cognitive decline. Nevertheless, further long-term follow-up studies are required to clarify the causal relationships. In addition, the lack of repeated assessments of serum copper level may have resulted in measurement errors due to diurnal variation (Guillard et al., 1979; Andersen et al., 2015). However, all blood samples for serum copper measurement were obtained at the same time of the day (08:00–09:00 a.m.) in all participants in order to minimize such errors.

## Conclusion

Our findings from non-demented older adults suggest that a low serum copper level within the normal range was associated with AD or cognitive decline via the moderating effect of iron. Regarding AD or cognitive decline prevention, clinicians need to pay more attention to avoid low serum copper and iron levels, even within the clinical normal range.

## Data Availability Statement

The study data are not freely accessible because the IRB of the Hallym University Dongtan Sacred Heart Hospital prevents public sharing of such data for privacy reasons. However, the data are available on reasonable request after IRB approval. Requests for data access can be submitted to an independent administrative coordinator by e-mail (yoon4645@gmail.com).

## Ethics Statement

The studies involving human participants were reviewed and approved by Institutional Review Board of the Hallym University Dongtan Sacred Heart Hospital. The patients/participants provided their written informed consent to participate in this study.

## Author Contributions

JK conceived, designed the study, and served as principal investigator and supervised the study. YC, G-HS, BL, I-GC, JL, HK, and JK were involved in acquisition, analysis and interpretation of the data, helped to draft the manuscript, and were major contributors in writing the manuscript, and critically revising the manuscript for intellectual content. All authors read and approved the final manuscript.

## Conflict of Interest

The authors declare that the research was conducted in the absence of any commercial or financial relationships that could be construed as a potential conflict of interest.

## Publisher’s Note

All claims expressed in this article are solely those of the authors and do not necessarily represent those of their affiliated organizations, or those of the publisher, the editors and the reviewers. Any product that may be evaluated in this article, or claim that may be made by its manufacturer, is not guaranteed or endorsed by the publisher.

## Footnotes

1
http://www.law.go.kr

2
https://www.mayocliniclabs.com
