# Supplementary material for: Association Between Copper and Global Cognition and the Moderating Effect of Iron
Source: Front Aging Neurosci. 2022 Mar 29;14:811117. doi: 10.3389/fnagi.2022.811117 (PMC9003994; doi:10.3389/fnagi.2022.811117)
Supplement: Supplementary file 1 [file Table_1.pdf]

SUPPLEMENTARY MATERIALS

Figure S1 Flow chart describing the composition of the GLAD study.

Table S1 Demographic and clinical characteristics of CN participants by the categories of serum copper

Table S2 Demographic and clinical characteristics of MCI participants by the categories of serum copper

This supplementary material has been provided by the authors to give readers additional information about their work.

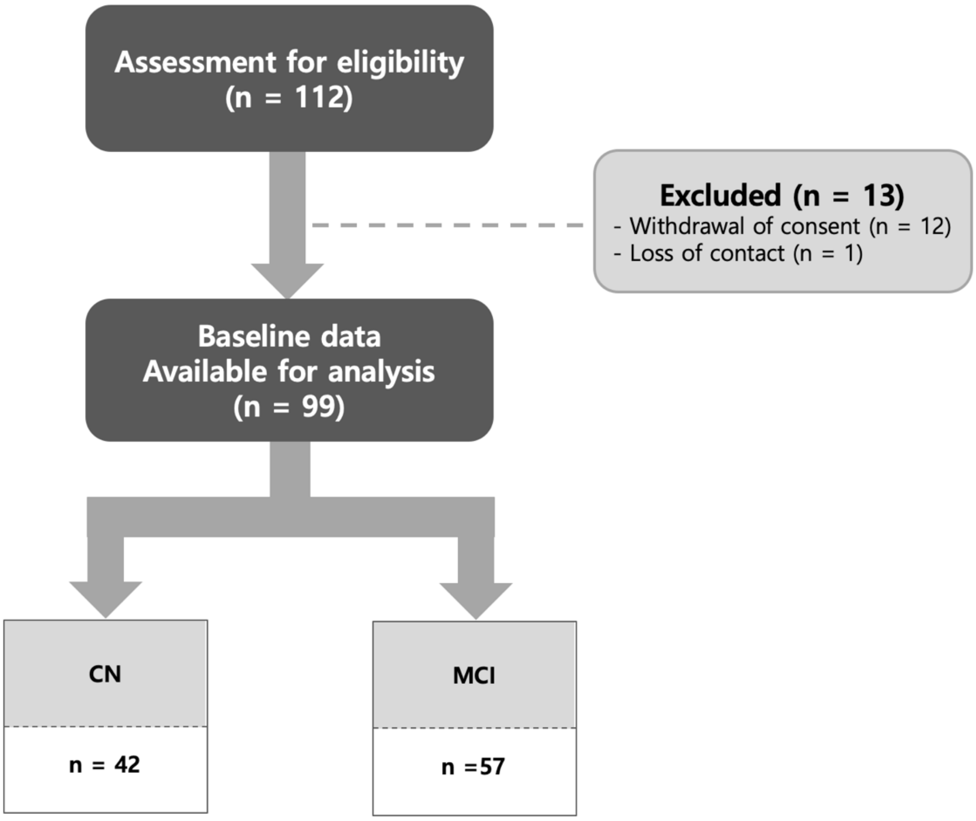

Figure 1. Flow chart describing the composition of the GLAD study.

Footnotes: CN = cognitively normal and MCI = mild cognitive impairment.

Table S1 Demographic and clinical characteristics of CN participants by the categories of serum copper

Characteristic	Overall	Categorized copper level			*P*
		Low	Medium	High	
n	42	11	19	12	
Age, y	72.38 (5.29)	72.09 (4.93)	72.53 (5.56)	72.42 (5.60)	0.977 [a]
Female, n (%)	33 (78.57)	8 (72.72)	14 (73.68)	11 (91.67)	0.504 [c]
Education, y	9.36 (4.79)	10.09 (5.03)	9.47 (4.46)	8.500 (5.35)	0.731 [a]
APOE4-positivity, n (%)	6 (14.29)	2 (18.18)	2 (10.53)	2 (16.67)	0.744 [c]
MCI, n (%)	0 (0.00)	0 (0.00)	0 (0.00)	0 (0.00)	
VRS, %	25.40 (15.72)	31.82 (13.85)	23.86 (17.84)	22.22 (12.97)	0.286 [a]
GDS score	8.88 (6.61)	8.09 (6.85)	9.58 (6.38)	8.50 (6.52)	0.812 [a]
Annual income status					0.509 [c]
<MCL, n (%)	2 (4.76)	1 (9.09)	0 (0.00)	1 (8.33)	
≥MCL, <2×MCL, n (%)	12 (28.57)	4 (36.36)	6 (31.58)	2 (16.67)	
≥2×MCL, n (%)	27 (64.29)	6 (54.54)	12 (63.16)	9 (75.00)	
Alcohol drink status, n (%)					0.702 [c]
Never	27 (64.29)	8 (72.72)	11 (58.89)	8 (66.67)	
Former	5 (11.90)	2 (18.18)	2 (10.53)	1 (8.33)	
Drinker	10 (23.81)	1 (9.09)	6 (31.58)	3 (25.00)	
Smoking status, n (%)					0.188 [c]
Never	35 (83.33)	10 (90.90)	14 (73.68)	11 (91.67)	
Former	6 (14.29)	1 (9.09)	5 (26.32)	0 (0.00)	
Smoker	1 (2.380	0 (0.00)	0 (0.00)	1 (8.33)	
Copper, ug/dL	93.93 (11.38)	81.82 (3.09)	92.11 (3.87)	107.92 (9.16)	<0.001 [a]
Hemoglobin, g/dL	13.49 (1.77)	13.56 (1.09)	13.47 (2.27)	13.45 (1.49)	0.987 [a]
Albumin, g/dL	4.58 (0.25)	4.64 (0.20)	4.60 (0.27)	4.51 (0.26)	0.449 [a]
Glucose, fasting, mg/dL	114.60 (26.84)	108.18 (18.99)	119.84 (32.29)	112.17 (23.59)	0.495
HDL-cholesterol, mg/dL	54.95 (10.70)	57.73 (12.54)	52.37 (9.14)	56.50 (11.18)	0.359 [a]
LDL-cholesterol, mg/dL	101.74 (33.74)	82.64 (21.10)	107.63 (28.47)	109.92 (44.87)	0.088 [a]
Iron, ug/dL	105.14 (31.77)	107.73 (25.66)	112.05 (35.40)	91.83 (28.82)	0.218 [a]
Iron					0.548 [b]
High (≥100 ug/dL), n (%)	21 (50.00)	4 (36.36)	10 (52.63)	7 (58.33)	
Low (<100 ug/dL), n (%)	21 (50.000	7 (63.63)	9 (47.37)	5 (16.67)	
Decrease in food intake over the past three months					0.459 [c]
no, n (%)	38 (90.48)	11 (100.00)	17 (89.47)	10 (41.67)	
yes, n (%)	4 (9.52)	0 (0.00)	2 (10.53)	2 (16.67)	
Global cognitive performance					
MMSE raw score	27.24 (2.10)	26.73 (2.72)	27.26 (1.82)	27.67 (1.97)	0.575 [a]
MMSE z- score	0.66 (0.73)	0.39 (0.82)	0.58 (0.80)	1.02 (0.29)	0.090 [a]

Abbreviations: APOE4, apolipoprotein E ε4 allele; MCI, mild cognitive impairment; VRS vascular risk score; GDS, geriatric depression scale; MCL minimum cost of living; MMSE, mini-mental state examination.

Data are expressed as mean (standard deviation), unless otherwise indicated.

[a] by one-way analysis of variance.

[b] by chi-square test.

[c] by fisher exact test.

Table S2 Demographic and clinical characteristics of MCI participants by the categories of serum copper

Characteristic	Overall	Categorized copper level			*P*
		Low	Medium	High	
n	57	21	16	20	
Age, y	72.16 (5.27)	72.62 (4.22)	72.56 (7.07)	71.35 (5.27)	0.719 [a]
Female, n (%)	43 (75.44)	17 (47.62)	13 (81.25)	13 (43.33)	0.505 [c]
Education, y	8.32 (4.48)	7.67 (3.41)	9.13 (5.24)	8.35 (4.93)	0.626 [a]
APOE4-positivity, n (%)	13 (22.81)	7 (33.33)	3 (18.75)	3 (15.00)	0.378 [c]
MCI, n (%)	57 (100.00)	21 (100.00)	16 (100.00)	13 (100.00)	
VRS, %	26.32 (20.64)	34.13 (18.62)	27.08 (24.25)	17.50 (16.64)	0.032 [a]
GDS score	12.56 (7.43)	10.24 (5.83)	12.19 (7.60)	15.30 (8.21)	0.088 [a]
Annual income status					0.498 [b]
<MCL, n (%)	16 (28.07)	7 (33.33)	2 (12.50)	7 (35.00)	
≥MCL, <2×MCL, n (%)	18 (31.58)	7 (33.33)	5 (31.25)	6 (30.00)	
≥2×MCL, n (%)	23 (40.35)	7 (33.33)	9 (56.25)	7 (35.00)	
Alcohol drink status, n (%)					0.510 [c]
Never	26 (45.51)	10 (47.62)	8 (50.00)	8 (40.00)	
Former	15 (26.32)	4 (19.05)	6 (37.50)	5 (25.00)	
Drinker	16 (28.07)	7 (33.33)	2 (12.50)	7 (35.00)	
Smoking status, n (%)					0.979 [c]
Never	43 (75.44)	15 (71.43)	13 (81.25)	15 (75.00)	
Former	12 (21.05)	5 (23.81)	3 (18.75)	4 (20.00)	
Smoker	2 (3.51)	1 (4.76)	0 (0.00)	1 (5.00)	
Copper, ug/dL	96.47 (16.89)	80.24 (3.62)	93.75 (3.07)	115.70 (11.54)	<0.001 [a]
Hemoglobin, g/dL	13.18 (1.51)	12.76 (1.92)	13.16 (1.19)	13.66 (1.15)	0.165 [a]
Albumin, g/dL	4.59 (0.26)	4.60 (0.25)	4.64 (0.27)	4.54 (0.26)	0.476 [a]
Glucose, fasting, mg/dL	111.75 (21.85)	112.10 (17.22)	103.81 (10.56)	117.75 (30.26)	0.164 [a]
HDL-cholesterol, mg/dL	55.16 (14.37)	52.00 (12.65)	63.44 (17.11)	51.85 (11.34)	0.022 [a]
LDL-cholesterol, mg/dL	94.51 (33.21)	92.57 (27.85)	92.94 (34.72)	97.80 (38.27)	0.863 [a]
Iron, ug/dL	97.70 (33.93)	93.14 (25.34)	93.38 (29.79)	105. 95 (43.64)	0.410 [a]
Iron					0.787 [b]
High (≥100 ug/dL), n (%)	29 (50.88)	11 (52.38)	9 (56.25)	9 (45.00)	
Low (<100 ug/dL), n (%)	28 (49.12)	10 (47.62)	7 (43.75)	11 (55.00)	
Decrease in food intake over the past three months					0.512 [c]
no, n (%)	47 (82.46)	17 (80.95)	12 (75.00)	18 (90.00)	
yes, n (%)	10 (17.54)	4 (19.050	4 (25.00)	2 (10.00)	
Global cognitive performance					
MMSE raw score	23.96 (4.14)	22.00 (4.02)	24.50 (3.48)	25.60 (4.08)	0.015 [a]
MMSE z- score	-0.42 (1.19)	-1.02 (1.27)	-0.29 (1.00)	0.12 (0.98)	0.006 [a]

Abbreviations: APOE4, apolipoprotein E ε4 allele; MCI, mild cognitive impairment; VRS vascular risk score; GDS, geriatric depression scale; MCL minimum cost of living; MMSE, mini-mental state examination.

Data are expressed as mean (standard deviation), unless otherwise indicated.

[a] by one-way analysis of variance.

[b] by chi-square test.

[c] by fisher exact test.
